# Lymphoplasmacytic variant of multiple myeloma

**DOI:** 10.1002/jha2.437

**Published:** 2022-04-08

**Authors:** Silje Johansen, Hilde Kollsete Gjelberg, Håkon Reikvam

**Affiliations:** ^1^ Department of Medicine Haukeland University Hospital Bergen Norway; ^2^ Department of Pathology Haukeland University Hospital Bergen Norway; ^3^ Department of Clinical Science University of Bergen Bergen Norway

**Keywords:** Lymphoproliferative disease‐ multiple myeloma

1

A 63‐year‐old previously healthy male was admitted to hospital with an intense cervical backpain. Magnetic resonance imaging (MRI) scan revealed osteolytic destruction and pathological fracture of vertebrae T1, and a low‐dose computer tomography scan showed no other sign of skeletal lesions. A weak monoclonal component, IgA, and increased kappa light chain to 82.4 mg/L (reference: 6.7–22.4) were detected in the patient's serum, and a bone marrow biopsy was performed. The results of histopathological examination of the bone marrow are shown in Figure 1. Hematoxylin‐eosin staining shows an expansion of cells, approximately 20%, with morphology similar to mature plasma cells admixed with smaller cells with scant cytoplasm, which thus showed more lymphoid morphology (A). The cells stained strongly positive for both plasma cell marker CD138 (B) and B‐cell marker CD20 (C), and were negative for CD45 (D) and PAX5. Kappa‐lambda staining showed kappa restriction (E). Noteworthy, cyclin D1 was strongly positive (F). Flow cytometry of the bone marrow showed following immunophenotype CD45 weak/38+/138+/19–/20+/56 weak and kappa intracellular light chains +. There were no other clinical myeloma features at diagnosis.

**FIGURE 1:**
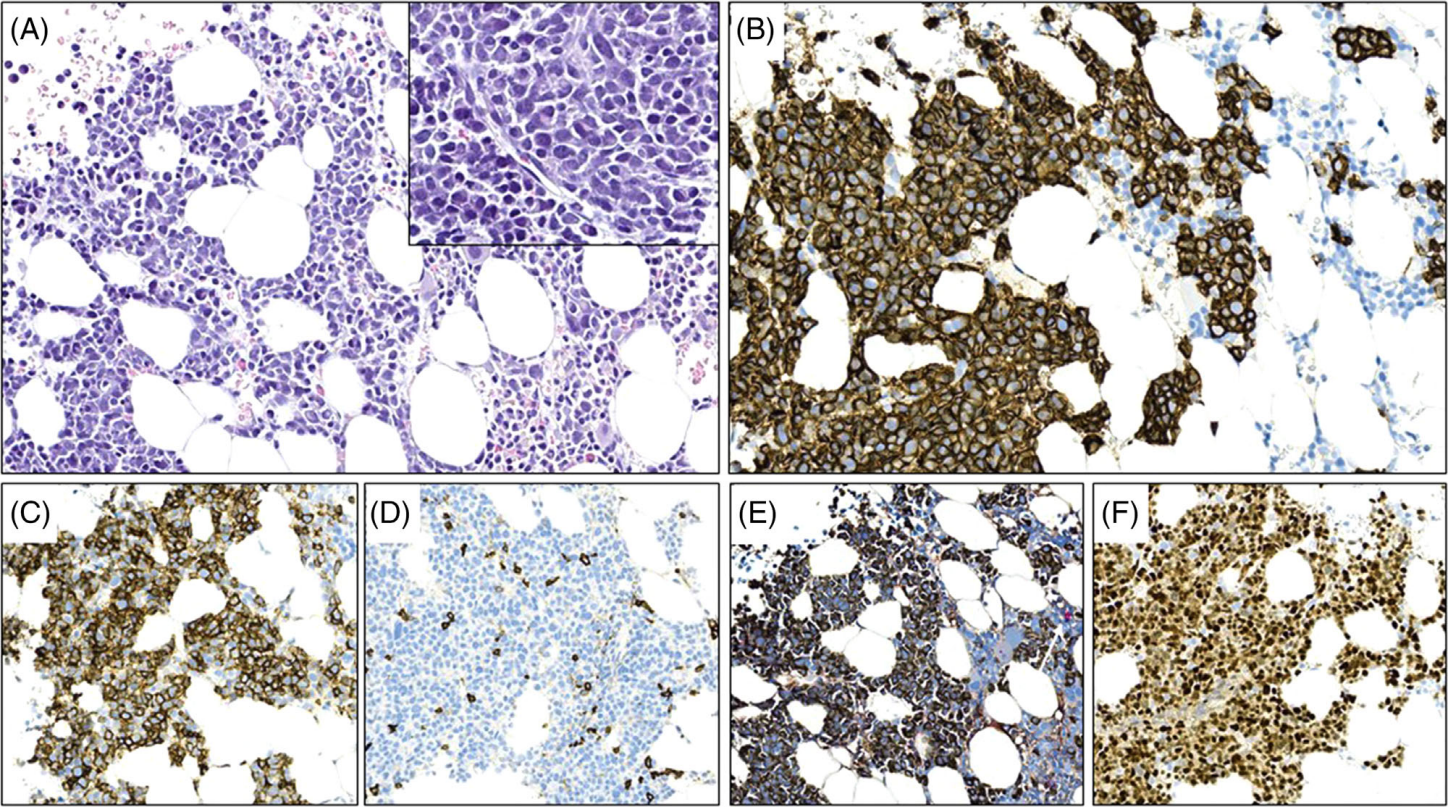
Histological examination bone marrow

These findings were considered to be compatible with lymphoplasmacytic variant of multiple myeloma (MM). The diagnosis was supported by fluorescence in situ hybridization (FISH) that confirmed the genetic abnormalities t(11;14) and 1q21+. He tested negative to t(14;4), t(4:16), and del17p, and screening for other somatic mutations was not performed. The patient was treated with initial radiation therapy. He was included in an ongoing study protocol for MM [1], where he received induction therapy with bortezomib, lenalidomide, and dexamethasone (Bort‐Len‐Dex‐regimen) repeated for four cycles. He was withdrawn from the study because of progressive disease with increasing kappa light chains and he quickly went to double autologous stem cell transplantation. He received a partial response after this treatment and is currently followed with lenalidomide maintenance therapy.

The traditional morphological diagnosis of MM has been based on morphology, an eccentric nucleus with spoke‐wheel chromatin, perinuclear court, and basophilic cytoplasm [2]. The traditional morphology examinations has been supplied with imunhistochemical examination demonstrating features of high CD38 and CD 138 expression [2]. In plasma cell dyscrasias, one typically finds a mix of CD45 positive and negative plasma cells. However, CD20 and cyclin D1 are seldom positive in MM in contrast to other lymphoproliferative neoplasia where cyclin D1 is positive, such as mantle cell lymphoma (MCL) and hairy cell leukemia. CD20 positive cells combined with the morphological picture indicates differential diagnosis such as B‐cell chronic lymphocytic leukemia (CLL) and lymphoplasmacytic lymphoma (LPL) [2]. These findings indicate that the malignant clonal expansion occurs in the transition between classical LPL and MM entity [3]. An association between the cytogenetic aberration t(11;14) has been described for this lymphoplasmacytic variant of MM [4]. The prognostic impact of this entity is mainly unknown, although the t(11;14) has been associated with better response to therapy and to some extend a better prognosis [4]. Hematologists and hematopathologists should be aware of this morphologic variant of B‐cells neoplasia, in particular differentiating it from other lymphoproliferative diseases [3].

## ETHICAL STATEMENT

Written informed consent for publication was obtained from the patient.

## References

[1] Rasmussen AM , Askeland FB , Schjesvold F . The next step for MRD in myeloma? Treating MRD relapse after first line treatment in the REMNANT study. Hemato 2020;1(2):36–48.

[2] Swerdlow SH , Campo E , Pileri SA , Harris NL , Stein H , Siebert R , et al. The 2016 revision of the World Health Organization classification of lymphoid neoplasms. Blood 2016;127(20):2375–90. 26980727 10.1182/blood-2016-01-643569 PMC4874220

[3] Hale DA , Krause JR . Plasma cell myeloma with lymphoplasmacytic morphology and cyclin D1 expression, an uncommon variant. Proceedings (Baylor University. Medical Center) 2017;30(2):192–4. 28405079 10.1080/08998280.2017.11929581 PMC5349825

[4] Lakshman A , Alhaj Moustafa M , Rajkumar SV , Dispenzieri A , Gertz MA , Buadi FK , et al. Natural history of t(11;14) multiple myeloma. Leukemia 2018;32(1):131–8. 28655925 10.1038/leu.2017.204

